# Intravenous Immunoglobulin and Immunomodulation of B-Cell – *in vitro* and *in vivo* Effects

**DOI:** 10.3389/fimmu.2015.00004

**Published:** 2015-01-22

**Authors:** Milica Mitrevski, Ramona Marrapodi, Alessandro Camponeschi, Filomena Monica Cavaliere, Cristina Lazzeri, Laura Todi, Marcella Visentini

**Affiliations:** ^1^Department of Clinical Medicine, Sapienza University of Rome, Rome, Italy; ^2^Department of Molecular Medicine, Sapienza University of Rome, Rome, Italy

**Keywords:** intravenous immunoglobulin, B lymphocytes, immunomodulation, CD21^low^ B-cell, autoimmune disease

## Abstract

Intravenous immunoglobulin (IVIG) is used as replacement therapy in patients with antibody deficiencies and at higher dosages in immune-mediated disorders. Although different mechanisms have been described *in vitro*, the *in vivo* immunomodulatory effects of IVIG are poorly understood. Different studies have suggested that IVIG modulates B-cell functions as activation, proliferation, and apoptosis. Recently, it was shown that IVIG induces *in vitro* B-cell unresponsiveness similar to anergy. In accord with this, we recently reported that IVIG therapy in patients affected by common variable immunodeficiency (CVID) interferes *in vivo* with the B-cell receptor (BCR) signaling by increasing constitutive ERK activation and by reducing the phosphorylated ERK increment induced by BCR cross-linking. Moreover, we observed that IVIG induces in CVID patients an increase of circulating CD21^low^ B-cells, an unusual population of anergic-like B-cells prone to apoptosis. Therefore, IVIG at replacement dose *in vivo* could prime B-cells to an anergic, apoptotic program. Here, we discuss these recent findings, which may improve our understanding of the immunomodulatory effects of IVIG, individualizing single involved molecules for more specific treatments.

Originally used only in primary and secondary immune deficiencies, intravenous immunoglobulin (IVIG), as a safe and efficacious therapy, has now increasingly been used for the treatment of different autoimmune and systemic inflammatory diseases (1). One of the most intriguing properties of IVIG preparations is the spectrum of interactions with our immune system involving almost all its components (2). The effects of these interactions [mediated by both the antigen-binding F(ab′)2 fragment and the Fc fragment of the IgG molecule] vary from one disease to another. In addition to antibodies against pathogens, a broad range of naturally occurring autoantibodies with the capacity to regulate important immune functions are present in IVIG. Despite the wide use of IVIG for immunodeficiencies as well as for various autoimmune and inflammatory disorders (indicated and off-label), the exact mechanisms of their immunomodulation remain not fully elucidated and somewhat controversial.

IgG antibodies are important for protecting us from microbial infections, but IgG autoantibodies are also major pathogenetic factors in several autoimmune diseases that benefit from IVIG therapy. Typical examples of these conditions are immunothrombocytopenia, autoimmune hemolytic anemia, and chronic inflammatory demyelinating polyneuropathy (CIDP). Therefore, the same class of molecules that promotes pathology in a disease can also be used as an anti-inflammatory treatment for the very same disease and this has been referred to as the intravenous IgG paradox (2).

B lymphocytes, unique cells with an immunoglobulin as a part of the B-cell receptor (BCR), are capable of interacting with IVIG in many ways. Our knowledge of these interactions is incomplete and largely based on *in vitro* experiments or on animal models, showing profound influence of IVIG on B-cell functions. The most important effects of IVIG on B-cells interfere with the fine balance of negative and positive signals, which maintain an appropriate B-cell activation threshold, critical for immune tolerance, and autoreactivity.

## IVIG and B-Cell Inhibitory Receptors Binding

Interaction of the BCR with the antigen results in signal transduction, which leads to the modulation of gene expression, resulting in activation, anergy, or apoptosis of B-cells. The role of co-receptors expressed on the B-cell surface is to modulate BCR signaling either positively or negatively. These co-receptors include the low-affinity receptor for IgG (FcγRIIb), CD22, and CD72, which negatively regulate BCR signaling, prevent overstimulation of the B-cells and are thus called inhibitory BCR co-receptors (3).

It has been shown that IVIG may interact with almost all these co-receptors significantly influencing B-cell fate.

IgG antibodies are glycoproteins that contain a carbohydrate moiety attached to each of the asparagine 297 residues in the two chains of the antibody Fc fragment. This glycan moiety is an integral structural component of the IgG molecule, forming part of the scaffold for FcγR binding. In addition, depending on the variable region sequences, nearly 20% of serum IgG antibodies have a F(ab′)2 fragment-attached N-linked sugar side chain (4). In 2006, Kaneko et al. for the first time demonstrated that IgG glycosylation and terminal sialic acid (SA) residues are crucial for IVIG activity in mice (5). Moreover, it was shown that only the enrichment of terminal SA residues of the Fc, but not of the F(ab′)2, fragments increased the therapeutic activity of IVIG (6).

These effects in B-cells are mostly mediated through the interaction of IVIG with CD22, a receptor belonging to the SA – binding Ig-like lectin (Siglec) superfamily. CD22 has seven immunoglobulin (Ig)-like extracellular domains and a cytoplasmic tail containing six tyrosines, three of which belong to the ITIM sequences. Unlike most other proteins from the immunoglobulin superfamily, Siglecs do not bind protein determinants but recognize exclusively sialylated carbohydrates. Sialylated glycans are usually absent on microbes but abundant in higher vertebrates and might therefore provide an important tolerogenic signal. CD22 plays a critical role in establishing signaling thresholds for B-cell activation. It is the dominant regulator of calcium signaling on conventional B2 lymphocytes (7). Séïté et al. proved that SA–IVIG colligation to CD22 promotes apoptosis via inhibiting the cascade of kinase phosphorylation in mature human tonsil B lymphocytes and in human Ramos lymphoma B-cell lines by inducing phosphorylation of ITIM (8). They also showed that only SA-positive IgG, but not SA-negative IgG bind to CD22, acting on several BCR-signaling pathways, including inhibition of the phospholipase Cγ2 cascade, sustained activation of extracellular signal-regulated kinases 1/2 (Erk1/2), p38, and down-regulation of PI3K. These changes are associated with the induction of cyclin-dependent kinase inhibitor p27Kip1, which inhibits cell-cycle progression at the G1phase and thus promotes apoptosis (8). Nevertheless, other authors, using CD22-deficient mice in models of ITP and K/BxN arthritis, could not demonstrate a role for CD22 in the immediate anti-inflammatory activity of IVIG (9).

FcγRIIb, another important B-cell inhibitory receptor, is a low-affinity single-chain receptor that carries an ITIM motif in its cytoplasmic domain, a hallmark of this inhibitory protein family. With the exception of T cells and NK cells, FcγRIIb is expressed on all cells of the immune system, and it is the only classical Fc receptor on B-cells. It regulates activating signals delivered by immunocomplexes retained on dendritic cells to the BCR (10). The inhibitory FcγRIIb on B-cells, by ITIM-dependent regulation of BCR signaling, is important in maintaining immune tolerance, thus preventing autoimmune disease. IgG immune complexes can colligate the FcγRIIb to the BCR, leading to inhibition of BCR-induced Ca2+ signals and cellular proliferation (11). It was demonstrated that FcγRIIb represents a checkpoint of human self-tolerance, probably during late stages of B-cell maturation (12). It was shown on murine and human cells that FcγRIIb controls bone marrow plasma cell persistence and apoptosis (13). Moreover, it was found that the isolated cross-linking of FcγRIIb on B-cells leads to B-cell apoptosis via ITIM- and SHIP-independent and c-Abl-family kinase-dependent pathways (14).

The study of Samuelsson et al. provided the first evidence that FcγRIIb is required for IVIG efficacy in mouse ITP (15). Animal models showed that *in vitro* and *in vivo* exposure of B lymphocytes from lupus-prone and from healthy mice to IVIG results in an increased expression of their surface inhibitory FcγRIIb receptors (16).

Tackenberg et al. found impaired inhibitory FcγRIIb expression on B-cells in CIDP with upregulation on monocytes and B-cells after clinically effective IVIG therapy, suggesting that strategies specifically targeting FcγRIIb might have therapeutic merit in this immune-mediated peripheral neuropathy (17). This is the first demonstration that IVIG *in vivo* results in the upregulation of FcγRIIB in human B-cells.

Recently, Bouhlal et al. showed that the existence of natural autoantibodies of IgG isotype directed against the FcγRIII and FcγRII. Interestingly, the immunopurified anti-FcγIII and anti-FcγII antibodies isolated from IVIG bind soluble and membrane bound FcR and inhibit rosette formation, suggesting that *in vivo* the natural anti FcR antibodies may inhibit the binding of immunocomplexes to the membrane receptors and interfere with the Fc-dependent functions (18).

## IVIG and Anti-Idiotypic Antibody Binding

Many antibodies to self-antigens are found in IVIG and are thought to have important role in its immunonodulatory effects. Some of these self-antigens include the variable domains of other antibodies and are recognized by anti-idiotypic antibodies (19), which may bind and neutralize pathogenic autoantibodies. An additional protective mechanism provided by anti-idiotypic antibodies is mediated through their binding by F(ab′)2 to the surface IgG or IgM of B-cells, transmitting negative signals and resulting in the downmodulation of pathogenic autoantibody production and elimination of potentially autoreactive clones (20, 21). Because of the small amounts of anti-idiotypic antibody in IVIG, it is not clear whether this potential mechanism of action of IVIG is a significant immune-modulating mechanism for autoimmune and inflammatory disorders. During the last decade, the beneficial effects of IVIG in diverse conditions were improved by using target-specific IVIG (sIVIG) *in vitro* and *in vivo* in animal models on such conditions as lupus (anti-DNA-idiotype-sIVIG in lupus mice) or antiphospholipid syndrome (anti-β2GPI-idiotype-sIVIG in APS mice) as a novel approach to treat different immune-mediated conditions in a more accurate antigen-specific manner (22). On a model of rats with experimental autoimmune myasthenia gravis and on blood samples from myasthenia gravis patients, Fuchs et al. demonstrated that a minor acetylcholine receptor-specific immunoglobulin fraction present in IVIG is essential for its suppressive activity (23).

An interesting mechanism of B-cell activation mediated by IVIG through a superantigen-like binding pattern was shown by Leucht et al. (24). They demonstrated that the favored Fab V_H_ germline gene segments bound by IVIG were 3–23 or 3–30/3–30.5, the most frequently rearranged V_H_ genes among human B-cells. In a subsequent study, they provided *in vivo* functional evidence, in patients with Kawasaki disease, that a subset of IVIG selectively activated B-cells of the same V_H_ germline origin, confirming the B-cell superantigen proprieties (25).

## IVIG Immunomodulation of B-Cell Antigen Presentation and Activation

In order to identify the cell surface molecules recognized by IVIG on human B-cells, Proulx et al. found that a significant amount of IVIG was spontaneously internalized by B-cells and interacted with intracellular targets, such as the lysosomal-trafficking regulator or nucleolin. They showed that IVIG internalization occurred in a BCR- and FcγR-independent pathway (26). More recently, the same authors demonstrated *in vitro* that IVIG in mice is able to inhibit the B-cell-mediated antigen-specific T cell activation following either BCR-dependent or BCR-independent antigen uptake. This inhibition of antigen presentation could not be explained by a modulation of MHC II molecules expression and was shown to occur in an FcγRIIb-independent manner, suggesting that the events responsible for the inhibitory effect occur at the intracellular level (27).

De Grandmont et al. observed that the addition of IVIG in culture of CD40L-stimulated B-cells reduced their expansion and stimulated the differentiation of part of peripheral B lymphocytes into IgG-secreting cells (28). The secreted IgGs were reactive with antigens such as nucleoprotamine, dsDNA, tetanus toxin, and human IgG F(ab′)2 fragments. Maddur et al. demonstrated that IVIG *in vitro* significantly inhibited the activation of BCR-stimulated B-cells and in a dose-dependent manner inhibited the proliferation of B-cells mediated by combination of anti-CD40 MAb, IL-21, and CpG (29). On the other hand, Heidt et al. showed that IVIG *in vitro* is not capable of directly inhibiting key B-cell responses, failing to affect the proliferative capacity of both purified *in vitro* stimulated B-cells and of autonomously growing B-cell hybridomas (30).

Thus, as with other immune-modulating activities of IVIG, these apparently contrasting observations may depend on the *in vitro* systems used and the state of activation of the B-cells exposed to IVIG (21).

An additional relevant mechanism of the immunoregulatory effects of IVIG in autoimmune disorders acts through the modulation of some toll like receptors (TLR) (31, 32). IVIG in culture, by using its Fc fraction, inhibits TLR-9 and TLR7-mediated B-cell activation and suppresses TLR-induced production of proinflammatory cytokines. IVIG mimics the effects of MyD88 inhibitor by suppressing TLR-induced B-cell activation and recruits the inhibitory SHP-1 phosphatase to regulate TLR-9 activation (33). Accordingly, Kessel et al. showed that IVIG attenuates the activation of TLR-9 and decreases secretion of IL-10 and IL-6 in B-cells from SLE patients (34).

## IVIG and B-Cell Anergy

Dussault et al. demonstrated on human B-cell lines that immunomodulation of human B-cells following treatment with IVIG involves increased phosphorylation of ERK and also Grb2-associated binder 1 and Akt, thus influencing BCR signaling (35). Other studies demonstrated that IVIG *in vitro* induces apoptosis of human B-cells through a Fas- and caspase-dependent pathway (36, 37). Besides this mechanism J. F. Séité and his group demonstrated that modulation of ERK activation in B-cells by IVIG ligation with CD22 is associated with cell-cycle arrest at the G1 phase and B-cell apoptosis (see above) (8). More recently, they showed that *in vitro* IVIG treatment of B-cells renders them refractory to BCR stimulation, suppresses the PI3K signaling pathway, and induces a long-term state of tolerance, promoting a program of long-term functional silencing similar to anergy (38). High-constitutive ERK phosphorylation is a central feature of murine models of anergy driven by constant BCR occupancy by antigen (39); in these anergic B-cells, constitutively activated ERK provides a tolerogenic signal dampening TLR-9 responsiveness (40). We showed that a subpopulation of human B-cells characterized by the reduced expression of CD21 (CD21^low^ B-cells) closely resemble murine anergic B-cells (41). CD21^low^ B-cells are expanded in a subset of patients with common variable immunodeficiency (CVID) and in some other immunological disorders and are characterized by high-constitutive ERK activation (41), low responsiveness to TLR-9 and BCR stimuli (42, 43), and propensity to apoptosis (44). In this regard, we observed that IVIG replacement therapy in CVID patients profoundly affects B-cell homeostasis (41). To investigate whether IVIG modulates *in vivo* ERK signaling in B-cells from CVID patients, we analyzed constitutive and BCR-induced ERK phosphorylation before and after IVIG infusion. We showed that unstimulated naive and IgM+ memory B-cells have significantly increased constitutive ERK activation after IVIG infusion, whereas BCR-induced activation, expressed as the fold increase respect to the constitutive ERK level, decreased in these cells (41). More recent observations from our group showed that IVIG infusion induces *in vivo* selective B-cell depletion in CVID patients. This effect is preceded by a profound modulation of B-cell homeostasis, where IVIG induces the down-regulation of CD21 expression promoting the generation of anergic-like, apoptosis prone CD21^low^ B-cells. We found that these newly generated CD21^low^ B-cells displayed the same peculiar pattern of receptors expressed by CD21^low^ B-cells present before IVIG, namely, increased FCRL4 and CD11c, and reduced CD62L expression (45).

In addition, these newly generated CD21^low^ B-cells, upon overnight culture, undergo spontaneous apoptosis. These observations suggest that IVIG therapy *in vivo*, even at a replacement dosage, may influence antibody responses by inducing B-cell depletion through differentiation into CD21^low^ B-cells that undergo accelerated apoptosis (45).

Bayry et al. demonstrated that IVIG *in vitro* at low doses induced proliferation and immunoglobulin synthesis from B-cells of CVID patients. It seems that IVIG rectifies the defective signaling of B-cells normally provided by T cells and delivers T-independent signaling for B-cells to proliferate (46). Moreover, in accord with our data, they showed that IVIG at low does induced the phosphorylation of ERK 1/2, Akt, and p38 MAPK in B-cells of CVID patients.

In conclusion, all these observations suggest that IVIG therapy in CVID patients, in particular, in those with autoimmune manifestations, may not only replace the missing antibodies but also regulate autoimmune and inflammatory responses through the modulation of B-cell functions (46).

## Interactions with B Lymphocytes’ Survival Factors

Intravenous immunoglobulin contains antibodies against many cytokines (47), but often the physiologic and therapeutic relevance of these antibodies remains unclear.

In humans, B-cell-activating factor (BAFF) is considered to be a master regulatory cytokine for B-cell homeostasis. BAFF serum levels are increased in a variety of B-cell related autoimmune disorders, like systemic lupus erythematosus (48), myasthenia gravis (49), and rheumatoid arthritis (50).

It was observed that natural antibodies present in IVIG could functionally neutralize cytokines, such as BAFF and proliferation-inducing ligand, important for B-cell survival (51). In fact, it was recently confirmed by two studies that IVIG treatment resulted in a significant decrease of BAFF serum level in newly diagnosed patients affected by CIDP, which all had elevated BAFF level before treatment (52, 53).

## Concluding Remarks

To date, a considerable amount of data on IVIG interactions with the immune system is available. However, most of them derive from *in vitro* or in animal models studies that do not completely reflect the pathophysiological status in clinical settings. It is therefore extremely important to correlate all these data in hand and to integrate them with *in vivo* studies on human disease. Moreover, no one single mechanism is responsible for the effects of IVIG in autoimmune diseases and immunomodulation on the different cells of the immune system should be combined for a better understanding of the therapeutic effects.

**Figure 1:**
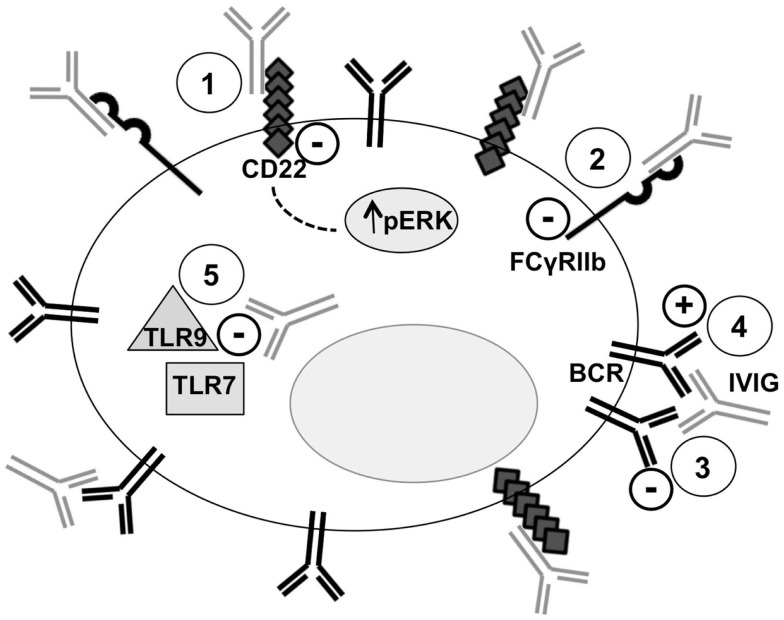
**Intravenous immunoglobulin and B-cell interactions**. Five mechanisms of the interactions between IVIG and B-cells are represented. *Point 1*: binding of SA–IVIG with the inhibitory receptor CD22 acts on several BCR-signaling pathways, including sustained activation of Erk1/2, promoting apoptosis. *Point 2*: binding of IVIG with the inhibitory receptor FcγRIIb leads to inhibition of BCR-induced Ca2+ signals and cellular proliferation. *Point 3*: anti-idiotypic binding of IVIG with the BCR transmits negative signals and modulates the production of pathogenic autoantibody. *Point 4*: B-cell activation is mediated by IVIG through a superantigen-like binding pattern. *Point 5*: IVIG inhibits TLR-9 and TLR7-mediated B-cell activation and suppresses TLR-induced production of proinflammatory cytokines.

B-cells play probably the most important role in the humoral immune response that, as demonstrated by the increasing amount of data, is profoundly affected by IVIG administration. A representation of some of the interactions of IVIG with B-cells is illustrated in Figure 1.

Despite many established aspects of IVIG–B-cell interactions, different other molecular mechanisms remain elusive. Increasing the knowledge of key molecules involved in the interaction of IVIG with B-cells may reveal which component of IVIG, whether, for example, a specific anti-idiotype antibody or an Ig fragment, is responsible for the immunomodulatory effects. This may provide the basis for the creation of more specific and tailored therapies for the different autoimmune diseases.

## Conflict of Interest Statement

The authors declare that the research was conducted in the absence of any commercial or financial relationships that could be construed as a potential conflict of interest.
